# Darwinian Agriculture: how understanding evolution can improve agriculture R. Ford Denison 2012. Princeton University Press

**DOI:** 10.1111/eva.12029

**Published:** 2013-01-24

**Authors:** Peter H Thrall

**Affiliations:** CSIRO Plant IndustryCanberra, ACT, Australia

Agroecosystems not only comprise a significant proportion of land-use, but also involve conflicting imperatives to expand or intensify production while simultaneously reducing environmental impacts. These imperatives are underpinned by food security concerns, climate predictability and global connectivity, reinforcing the likelihood of further major changes in agricultural landscapes and associated production systems in coming decades. These changes are likely to include adoption of novel genetic technologies and agronomic practices, shifts in patterns of land-use and perhaps even new crop species. Ford Denison's new book, *Darwinian Agriculture: how understanding evolution can improve agriculture*, makes a strong and very personal case for the application of evolutionary principles to addressing the twin challenges of feeding an expanding human population while working to reduce agriculture's environmental footprint. Of course, the use of eco-evolutionary principles is not new in agriculture (e.g. crop breeding and management of selection for pest resistance), but given land-use trends and other transformative processes in production landscapes, ecological and evolutionary research in agroecosystems must consider such issues in a broader systems' context (Thrall et al. 2011). I fully agree with Denison that evolutionary concepts have potential to help us deal more effectively with these complex problems and that multidisciplinary approaches are needed to improve both productivity and sustainability.

*Darwinian Agriculture* focuses primarily on one particular aspect of this broad topic, and that is to do with the opportunities and challenges around producing more food. Fair enough! Meeting projected food demands over the next several decades could prove to be a more difficult challenge than climate change itself. Denison uses this central focus to draw in discussions of a diverse and somewhat idiosyncratic but interesting range of subjects (e.g. leaf-cutter ants, natural selection and the evolution of simple vs. complex traits, his brother Tom's organic farm, kin selection and male sterility in corn). This seemingly disconnected set of topics is used to variously illustrate points within three central themes that permeate the book. Firstly, Denison argues that the concept of trade-offs is absolutely critical. This is tightly connected with his second major theme, which is that any simple evolutionary advances (i.e. trade-off free) are likely to have already been tried and tested via natural selection. He suggests that further direct ‘improvements’ in species performance (e.g. drought tolerance and perenniality) are generally likely to come with costs. Related to this is his view that, while there may be increasingly limited potential to improve individual traits, there is likely to have been much less optimisation at the community level, so there may be more possibilities for utilising eco-evolutionary principles (Chapter 1). In this context, Denison makes the useful point that improving agriculture may be more of a group selection problem (e.g. Chapters 4, 8), and this also relates to the scarcity of ‘low-hanging’ individual traits. Examples include crops that could better compete with weeds, solar tracking or among-species cooperation, for example, with mutualists (Chapters 8, 9). Finally, a third theme that is explored in several contexts is the extent to which we can use natural systems as a model for agriculture, and if so, what components of natural systems could most usefully be transferred to modern agroecosystems. These themes are developed early on and returned to throughout the rest of the book.

In many ways, I enjoyed reading this book. We need all the ideas on this topic we can get – maintaining global food security while protecting our natural ecosystems is not going to be easy! I especially like the goal of developing a ‘unified field-crop theory’ (Chapter 4). Overall, Denison does a good job of covering the basic issues in agriculture (population increases, food demand trends, input costs, etc.) that argue why we need an eco-evolutionary perspective for longer-term sustainability (Chapter 2). He also highlights the need for innovative thinking around the challenges of food security and improving the sustainability of our farming systems. While I can't say I agree with everything he says (more on that below), the book has stimulated plenty of discussion with colleagues and that is certainly worthwhile. It is also an entertaining journey through Ford's personal perspective on the concept of Darwinian agriculture – something he has clearly thought about for some time.

At the same time, it is not 100% obvious to me who the intended audience is. Do scientists really need to be convinced that ecosystems don't behave like species (who would be likely to debate such a statement)? Overall, the book is written in what I would call a semi-scientific and rather anecdotal style; there is a bibliography, but individual statements are not consistently referenced as would be the case in a more traditionally academic text. Much of what is written seems more like subjective opinion than documented facts. For example, early on in Chapter 6, Denison states that ‘The near-perfection of natural ecosystems is apparently the foundational hypothesis for many scientists who call themselves agroecologists’ and later that ‘typically agroecologists may choose more diverse systems to copy’. I'd have to say that this doesn't represent the majority of scientists I know who work in agroecological systems. Are the agroecologists that Denison refers to working at the ‘more organic’ end of agronomy? The only reference to the first statement is by Gutierrez and Daxl (1984). I think the world has moved on, at least within the contingent of scientists who are seriously trying to use eco-evolutionary principles to investigate questions in agroecosystems. For example, there are increasing numbers of researchers focused on issues such as understanding how agricultural landscape structure influences insect pest and predator abundances, how the spatial arrangement of crops influences disease dynamics and evolution, or exploring how agronomic management practices impact on soil biota. So at least in the context of understanding and managing biotic interactions, I would argue that agroecologists generally aim to use (not copy) principles derived from studies of natural ecosystems.

To a significant extent, Denison appears frequently not to be a fan of biotechnology (e.g. Chapter 5) or is at least quite sceptical. I don't have a problem with this at all, but there is an implication here that the results reported by scientists working for biotechnical companies are perhaps less objective than, for example, the views put forward by the Union of Concerned Scientists – I'm not so sure that this is fair. This is not to say that Denison doesn't recognise some of the advances made through such approaches. In point of fact, there have no doubt been many overstated claims of likely progress and benefits to be derived from genetic engineering, and I'm not sure how many spectacular examples of success there are, at least with regard to directly improving crop yield. However, this doesn't mean that biotechnologists are completely blind to the potential constraints of trade-offs (Chapter 4), which appears to be one issue that Denison has with biotechnology, and there can be broader environmental benefits (e.g. reduced pesticide use in Bt cotton fields). In fact more generally I'd suggest that many agronomists at least implicitly already think about trade-offs. Thus, when breeders trial new crop varieties, they usually consider other traits as well as yield, including growth in different environments, disease resistance, performance under different management regimes, etc. Agronomists also largely focus on raising collective productivity (e.g. field or farm level differences in yield), so one could argue that this represents some underlying notion that group properties are more important than individual performance.

Denison's concerns here are partly related to the concept I mentioned earlier, namely that the ‘easy’ adaptations would largely already have been evaluated by natural selection over millions of years. I'm not sure this is an entirely valid argument. Just because traits might have been rejected by natural selection doesn't mean that they aren't worth trying in the context of modern agricultural systems. These represent qualitatively different situations from nature that might favour quite different trait combinations. In fact, Denison clearly recognises this, as he points out, natural systems are different from agricultural systems so we shouldn't necessarily try to imitate them.

Examples that Denison specifically discusses (Chapter 5) with regard to the challenges of genetic engineering new traits in crop species include improving nutrient use and water use efficiency. Is it really true that there are no gains to be made here? There is certainly a lot of work going on around the world in this regard. Similarly, he spends considerable time on global initiatives to develop C_4_ rice plants that might have real photosynthetic benefits in some environments. I can well believe that this is an extremely difficult multigenic challenge. A recent *Science* article (von Caemmerer et al. 2012) suggests that they ‘expect to have a C_4_ rice prototype within 3 years’ but also recognises it will take longer to optimise and field test. Denison isn't convinced, but it seems to me that the very fact that C_4_ photosynthetic pathways evolved so many times in so many different plant groups suggests that it may be less difficult than he thinks (of course natural selection has had a very long time to work on the problem…). Overall, I think that if the risks of using biotechnology are small relative to the potential benefits, then we should persist (even if it takes awhile to get there). After all population growth, the need for sustainable farming systems as well as global food security is not going away any time soon, and it often isn't easy to predict what will work. It might be worth noting in this context that no-till farming was proposed back in the 1960s but took several decades to catch on even without the difficult regulatory issues and other constraints faced by genetic engineering.

A considerable portion of the book, motivated by a paper by Jackson and Piper (1989), is devoted to making a case for why we shouldn't just blindly copy natural systems in the process of improving agricultural sustainability and productivity (e.g. Chapters 6–8). While I don't disagree with this, I am also pretty sure that neither would most agroecological scientists and production agronomists so I am not sure who the argument is intended for. Clearly, natural ecosystems are not organised in ways that would maximise food production for humans. In this sense, I didn't find Denison's comparisons of production in natural and agricultural systems particularly enlightening (Chapter 6). This doesn't mean that we can't learn from natural systems; many of the questions that agroecologists are beginning to address from a broader community-based perspective are explicitly based on prior understanding acquired from work in unmanaged ecosystems: what kinds of plant communities are likely to be more resistant to weed invasions? Is it possible to build pest-suppressive agricultural landscapes? How do crop pathogens evolve in relation to the spatial arrangement and diversity of crop cultivars? How does agronomic management influence soil community structure and function (e.g. in the context of disease suppression or symbiotic mutualists)?

In summary, while the major impacts of climate change on our landscapes and ecosystems are probably yet to be felt, other drivers such as food security and global connectivity are already leading to shifts in land-use patterns and environmental impacts, placing pressure on agricultural production systems. While one could quibble about the exact numbers, projected population increases indicate that global crop production will have to increase by at least 70% by 2050 (Tilman et al. 2011). Dealing with this global food demand will require a combination of approaches, including the following: a) reducing demand; b) filling the production gap via, for example, expanding the amount of arable land, agricultural intensification and diversification, or raising yield potential; and c) avoiding production losses (e.g. through reducing climate change impacts, avoiding land/water degradation, effective management of biotic threats and maintaining valuable ecosystem services such as those provided by soil biota). Denison's book touches on many of these but largely through the lens of increasing production and the need to consider constraints associated with trade-offs. While he does discuss biotic interactions (e.g. evolution of pest and weeds, biological control; Chapters 10, 11), this is not the central aim of the book, in part because he suggests that directly increasing yield potential is more critical. I don't argue with the importance of yield increases, but I think achieving sustainable food production goals of the magnitude predicted will require all the tools at our disposal.

At the end of the day, *Darwinian Agriculture* provides an interesting and passionate but rather personal perspective that certainly challenges us to think a lot harder about what eco-evolutionary principles might have to offer agriculture (and *vice versa*), and it will hopefully stimulate a lot more scientists to conduct research across the agroecological interface (Chapter 12). I don't agree with everything in it, and there are many other topics that could have been included, but it has certainly got me thinking, and that is really all one can ask of a book such as this.
